# Defining vulnerabilities and enabling community engagement in epidemics preparedness: the CAVE model from Austria

**DOI:** 10.1093/eurpub/ckae173

**Published:** 2024-11-08

**Authors:** Paul Grohma, Silvia Wojczewski, Barbara Juen, Priya-Lena Riedel, Frederik Seufert, Vanessa Streifeneder, Steffen Reichel, Sandra Pichler, Vanessa Kulcar, Sandra Nestlinger, Monika Stickler, Cornelia Schober, Hermann Scheller, Ruth Kutalek

**Affiliations:** Unit Medical Anthropology and Global Health, Department for Social and Preventive Medicine, Center for Public Health, Medical University of Vienna, Vienna, Austria; Department for Primary Care Medicine, Center for Public Health, Medical University of Vienna, Vienna, Austria; Institute for Psychology, University of Innsbruck, Innsbruck, Austria; Institute for Psychology, University of Innsbruck, Innsbruck, Austria; Institute for Psychology, University of Innsbruck, Innsbruck, Austria; Department of Geoinformatics Z_GIS, Paris Lodron University of Salzburg, Salzburg, Austria; Department of Geoinformatics Z_GIS, Paris Lodron University of Salzburg, Salzburg, Austria; Spatial Services GmbH, Salzburg, Austria; Disaster Competence Network Austria (DCNA), University of Innsbruck, Innsbruck, Austria; Institute for Psychology, University of Innsbruck, Innsbruck, Austria; Disaster Competence Network Austria (DCNA), University of Innsbruck, Innsbruck, Austria; Austrian Red Cross, Vienna, Austria; Austrian Red Cross, Vienna, Austria; Lebenshilfe Tirol, Innsbruck, Austria; Safe Reach GmbH, Vienna, Austria; Unit Medical Anthropology and Global Health, Department for Social and Preventive Medicine, Center for Public Health, Medical University of Vienna, Vienna, Austria

## Abstract

During the COVID-19 outbreak the transdisciplinary research project CAVE (Community Engagement and Vulnerability Assessment in Epidemics) investigated perceptions and practicability of public health communication among stakeholders of care and social facilities in Austria. It aimed at finding accurate definitions of vulnerability in the context of epidemics and at developing operational models for engagement of vulnerable groups in co-creating epidemic response mechanisms. Transdisciplinary methods included semi-structured interviews, focus group discussions, and desk reviews as well as spatial analysis and composite indicator-based mapping methods. Informants and participants in the community engagement phase represented clients as well as middle and lower management levels of Austrian care and social facilities for older persons and persons with cognitive impairments, persons depending on mobile healthcare services, homeless people, and socially deprived communities. A narrow definition of vulnerability as well as missing strategies for participation and community engagement limited communication with stakeholders and the implementation of protective measures. An inclusive definition of vulnerability beyond medical and physical indicators should be employed to account for social, psychological, and emotional aspects contributing to a higher risk of being affected by epidemics. The CAVE model provides a multi-level definition of vulnerability that allows for participatory engagement in co-creating adapted crisis response measures. We suggest policymakers and health authorities to consider a broader definition of vulnerability and to commit to pro-active engagement with stakeholders representing these sectors. This requires the establishment and maintenance of communication structures as well as political recognition of civic participation in the creation and implementation of epidemic response measures.

## Introduction

The project CAVE (Community Engagement and Vulnerability Assessment in Epidemics) aimed at finding operational definitions of the two central terms vulnerability and engagement, and at producing a tool (model) to support policymakers and civil society organizations (CSOs) to address and integrate vulnerable populations before, during and after infectious disease outbreaks.

The project was built on three explorative qualitative surveys among diverse social and care organizations for vulnerable populations across Austria [1–3] asking about their employees’ and clients’ experiences with, at that time still valid COVID-19 regulations, regarding their adequacy and room for maneuver. The organizations surveyed include small- and medium-sized care facilities for older persons, mobile healthcare services (including psychiatric care), services for people suffering from physical and cognitive impairments, homeless people, and counseling services for socioeconomically deprived people.

The surveys indicated that the definitions applied by Austrian health authorities to describe vulnerability, as well as the methodology to include vulnerable populations in pandemic response, were only partly appropriate to cover all groups that were severely affected by the disease. Risk communication and protective measures largely focused on medically determined vulnerabilities, leaving aside individuals’ and groups’ exposure due to social disadvantages or cognitive impairments. The surveys also revealed a need to establish and increase the functionality of communication structures between CSOs and public health authorities, as well as within support organizations.

**Figure 1.**
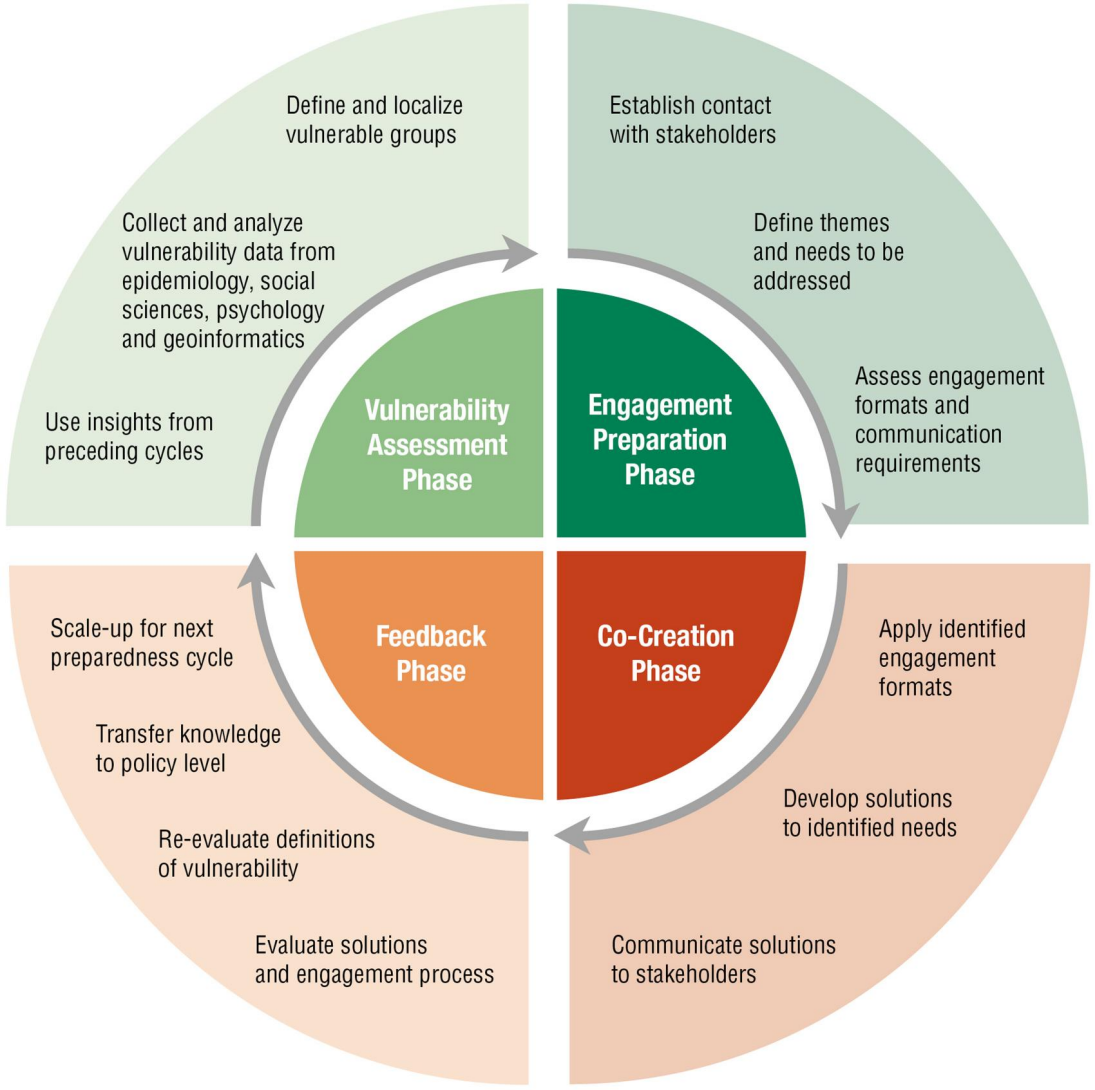
The CAVE model.

Based on these findings, the CAVE project produced a definition that accounts for different levels of vulnerability (see Table 1) and a model for community engagement in crisis management that suggests a cycle of definition, localization, inclusion, and long-term collaboration with diverse risk groups and under-represented populations (see Fig. 1). Both, the definition of vulnerability and the CAVE model are supported by geoscience data, helping to determine geographical target areas, as well as regionally varying socio-economic risk factors that contribute to increased vulnerability [4, 5]. Two results of the CAVE project, the definition of vulnerability and the CAVE model for community engagement in epidemics, which were tested in practice during and after COVID-19, are described in this article.

**Table 1.** Levels of vulnerability during epidemics

	Indicators	Group of persons/communities	Community engagement (meta-)themes	Community engagement formats/examples from CAVE
Primary vulnerability	Physical and medical indicators (advanced age, pre-existing illness, professional contact with patients)	Hospitalized persons, chronically ill, clients of nursing homes, and hospital staff	Dismantling hierarchical structures Creation of participatory channels Involvement of relatives and multiple staff levels	Moderated discussions/focus groups between management and staff Workshops/group events with clients and relatives Workshops for creating communication apps (notification systems; interactive apps)
Secondary vulnerability	Limited access to health information	Persons with limitations in cognitive functioning Persons with low levels of education Persons with a history of migration	Translation of public health messages and decrees into the context of the client/care facility Translation into simple language Translation into multiple languages	Round tables/direct contact with authorities and CSOs (identification of challenges, needs and resources) Low-threshold platforms for information exchange; Games-based mediation Involve peer ambassadors from specific communities
Tertiary vulnerability	Health crisis caused by epidemic response measures (consequences of lockdown and social distancing)	Persons depending on services that are no longer available in an epidemic (live-in care, psycho-social support, persons living with disabilities) Socially disadvantaged groups who rely on welfare (homeless, women’s shelters, food banks, emergency shelters, outpatient medical care) Persons who are cut off from social contacts and suffer from the psychological consequences	Development of concepts for the reduced continuation services (situational adaptation of measures) Development of concepts for maintaining reduced contact (information on protection; psychosocial support) Development of concepts for psychosocial support	Round tables/direct contact with authorities and CSOs (identification of challenges, needs and resources) Workshops (CSO management, staff, authorities) to maintain minimal operation of social services Development of concepts for the integration/creation of communication channels and psychosocial support offers

Alt text: The table shows three distinct levels of vulnerability during epidemics, broken down by indicators, target groups, engagement themes, and engagement formats. © Medical University of Vienna.

## Methods

The definition of vulnerability and the CAVE model are based on the findings of consecutive project phases. In the research phase, a mixed methods approach was applied, including qualitative surveys among CSOs and clients of care and support organizations to collect opinions on established concepts of vulnerability and the perception and practicability of the Austrian public health approach to risk communication. We conducted 21 semi-structured online interviews with CSOs in eastern, southern, and northern Austria, focusing on middle and higher management levels of a large variety of organizations. Additionally, we conducted 11 online-based guided interviews and eight focus groups in care facilities and assisted living accommodations with clients, staff, and lower management building on previous research in similar settings in Austria, Germany, and Southern Tirol [1]. Interviews lasted between 35 and 80 min, and focus groups between 45 and 90 min. Interviews and focus groups were recorded, transcribed, and pseudonymized with informed consent of all participants. The transcripts were analyzed using Atlas.ti software and content analysis [6]. For coding and interpretation of data, the UNICEF minimum standards and indicators for community engagement [7] were consulted to describe the degree of participation, inclusion, empowerment and ownership, two-way communication, adaptability, and localization, as well as building on local capacities. These surveys were supported by a desk review to identify existing concepts of vulnerability [8–10], resilience [11], intersectionality [12], and participation [13, 14], as well as crisis management models [15–19] that can be used to engage communities in outlining their adequate risk communication and response strategies. These findings determined the formation of the socio-economic vulnerability assessment tool [4], the CAVE model for community engagement, and the parameters for a subsequent community engagement phase. The socio-economic vulnerability assessment tool is based on spatial analysis and composite indicator-based mapping methods that integrate social science findings into an interactive virtual map (GIS tool). This map highlights geographic regions in Austria with elevated vulnerabilities, which can be filtered in accordance with variable indicators as, e.g. distance to care facilities, age structure, socio-economic structure, proportion of migrant communities, and more. A detailed description of the map and the spatial analysis and composite indicator-based mapping method can be found in Streifeneder *et al*. [4].

After the definition of an operational term for vulnerability and the creation of the CAVE model, these results underwent a test in four of our project partners’ care facilities (Austrian Red Cross and Lebenshilfe Tirol), where different crisis scenarios were played out in engagement processes with clients and different levels of facility staff. This implementation phase was scientifically accompanied [20] and fed into the final output of the project, including recommendations for policymakers, social and care organizations, and emergency services (ambulances, fire departments, mobile support units, crisis intervention teams).

## Results

### The CAVE definition of vulnerability

The findings of research phase [3] suggest that during the early phase of the COVID-19 pandemic in Austria, the concept of vulnerability was used in a limited scope, largely reducing the term to ‘physical vulnerabilities’ such as advanced age, pre-existing illnesses or exposure due to professional contact with patients. At a later stage of the pandemic, public health authorities added persons with mental disorders and limitations in cognitive functioning, such as dementia and intellectual disabilities, to the officially recognized risk groups. However, the definition did not consider groups and individuals showing features of social vulnerability like poverty, social marginalization, or mental stress that were caused by the outbreak and its response measures. Survey results indicate that public health messages and regulations directed at the official risk groups were perceived as not reflecting the varying demands of clients and as inconsistent with working routines of caregivers. Respondents clearly supported a simplification of pandemic regulations in terms of an adaption to the diverse contexts of vulnerable groups. Such adjustments can be addressed with community engagement processes that take into account the variety and complexity of vulnerable groups, their demands, and their capacities.

Therefore, the CAVE definition discerns different levels of vulnerability considering social, political, economic as well as psychological and cultural factors in addition to merely “physical vulnerabilities.” Aside from medical indications, the definition accounts for vulnerabilities caused by limited access to information, e.g. due to cognitive limitations or to social and economic challenges, such as language barriers, illiteracy, and lacking access to online devices. The third level of vulnerability represents groups with increased vulnerability caused by epidemic response measures, such as contact restrictions, lockdowns, and social distancing.

Our qualitative research revealed significant additional challenges for people depending on live-in caregivers and day-care centers or on contact persons for psycho-social support, when services were no longer accessible or switched to online assistance. In addition, a severe gap in psychological support for children and adolescents became apparent demonstrating the need for improved concepts of engagement for this level of vulnerability [3].

Next to the description of vulnerability indicators and vulnerable groups, Table 1 includes a column with engagement (meta-) themes that were gathered during the CAVE community engagement phase. These themes were carved out in engagement processes with multi-level stakeholders of care organizations who co-created adapted risk management concepts for their facilities [2, 20]. The core issues identified with high priority are assigned to the respective levels of vulnerability and social groups. The last column presents examples of formats to implement engagement processes in accordance with specific groups and themes.

### The CAVE model

The CAVE model for engagement with people in vulnerable situations caused by epidemic events grounds on the results of the research phase and was subsequently tested in the community engagement phase in different settings of our CSO partners’ care and support facilities [20].

The structure of the model is based on the ECDC’s guidance on community engagement for public health events caused by communicable disease threats in the EU/EEA [15], which offers instructions to include communities in pandemic management. It refers to a preparedness cycle that distinguishes the three phases of anticipation, response, and recovery. Similarly, the OECD proposes such a three-stage cycle as a framework for evaluations of COVID-19 measures [14]. The CAVE model combines this cyclical understanding of crises [21] with multi-stage crisis management models [22–24], which underline pre- and post-crisis phases as important learning processes to inform the development of response measures. The CAVE model represents a cyclical risk management model that understands health crises (infectious disease outbreaks) as recurring events, the findings of which should be integrated into long-term and continuous preparation for future crises (Disease X).

In contrast to the approach of ECDC and OECD, the CAVE model envisages four phases in the crisis management cycle, with two preparation phases dedicated to the definition and localization of vulnerabilities, as well as to the establishment of long-term cooperation structures between vulnerable groups and related stakeholders, thus reflecting the deficits identified through qualitative research.

The CAVE model corresponds with the multi-level definition of vulnerability and includes a separate phase to assess and cyclically re-assess certain groups’ vulnerabilities. This assessment phase (1) aims at a broader understanding, systematic differentiation, and contextualization of vulnerabilities in order to identify and localize specific groups and their relevant challenges. Epidemiological, social science, and geographical data are used to determine groups or regions with elevated vulnerability and to analyze their characteristics. This phase serves to understand certain groups’ demands and specifics, their social, geographical, and socio-economic situation, as well as their forms of organization and representation. The vulnerability assessment informed the CAVE community engagement phase and delivered the data basis for a GIS tool to localize and visualize vulnerable communities in Austria [4].

The engagement preparation phase (2) is dedicated to establish or deepen the relationship between stakeholders relevant in a crisis and to develop adequate and contextualized themes to be addressed in engagement processes that relate to their demands, resources, and capacities. Phase two underlines the importance to create and foster a solid, long-term basis for dialogue between decision-makers and stakeholders, which are currently missing in Austria’s risk communication strategy and in existing crisis management models. Established collaboration with representatives of vulnerable groups facilitates the designation of issues to be addressed and the notification of needs for adjustments to publicly decreed health regulations. This phase is also necessary to know a specific group’s communication requirements, as e.g. the use of simple language or accessibility of technology. Both preparation phases (1 and 2) serve to anticipate recurring health crisis and account for a cyclic production of knowledge that feeds into the definition of vulnerability and determines certain groups’ response needs and capacities based on continuous research and experiences from past crisis. They should not be seen as punctual interventions but as constant processes requiring resources of time and personnel over a long-term period.

The CAVE project identified people in care (nursing and welfare) facilities, people depending on live-in care or mobile assistance, and people with physical and/or cognitive impairments as vulnerable groups that had difficulties to cope with the public COVID-19 risk communication and expressed demands to adjust epidemic regulations to their contexts. During the community engagement test, our CSO partners negotiated with stakeholders (clients, caregivers, technical staff), which crisis scenarios are feasible to be addressed within their facilities, and which engagement formats are constructive for their clients to work on solutions.

The co-creation phase (3) follows the same principles of participation, and it aims at determining relevant and actionable crisis response measures. The themes identified in phase two are addressed with engagement formats that were agreed as adequate. Topics like, e.g. the improvement of communication structures within care institutions or translations and contextualization of risk regulations can be facilitated with a variety of participatory techniques (see Table 1). Solutions can be workshops with clients and stakeholders to adjust epidemic regulations, the creation of social spaces, digital communication tools or concepts for visitors, and for inclusion of relatives that reflect the needs and capacities within care and support organizations. Phase three takes effect when the specific disease is already known and relevant for public health measures. The co-creation of risk communication and response strategies requires detailed knowledge about the pathogen and its transmission. According to the diverse needs and capabilities elaborated in the preparation phase (2), the CAVE community engagement delivered a great variety of co-created solutions, including emergency plan adaptations to epidemic conditions and a mobile alarm app to call in dependents in case of an epidemic caused shortfall of personnel.

The feedback phase (4) is intended to transfer knowledge from a past crisis and to update definitions of vulnerability for the next cycle of preparedness. Co-created solutions to epidemic challenges as well as the engagement process itself should be evaluated with regard to adequacy, inclusion, understandability, practicability, and contextualization. Goals and engagement formats might need to be changed or further adapted if they did not deliver the expected outcomes. This phase also serves to look back on the applied definition of vulnerability with regard to its accuracy in correlation with past epidemic events. An important part of the feedback phase concerns the transfer of knowledge to the political level in order to benefit a larger number of vulnerable stakeholders in the next cycle. Systematic documentation of processes and results as well as translating findings into communicable content for policymakers are vital for scaling up solutions that were successfully developed and implemented in individual case studies or research projects.

## Discussion

Literature reviews on the concepts of vulnerability and community engagement suggest that “social capital, fundamental cause theory, social determinants of disease, and other concepts such as structural violence offer not only interesting theoretical understandings of the connections between vulnerability and community engagement, but also provide a potential theory of change for those who wish to use either concept as an intervention in the context of infectious diseases” [8]. Especially disaster studies [5, 12, 25] but also medical anthropology studies [26] already make use of descriptions of social vulnerability that highlight some historical, cultural, and ecological framings for understanding vulnerability [8]. With this understanding in mind, the CAVE project aimed at translating the connectivity of vulnerability and community engagement into practice in Austria, and to deliver support to policymakers and CSOs in addressing and integrating vulnerable populations before, during, and after infectious disease outbreaks.

While advocating for a more differentiated and inclusive definition of vulnerability we realize that medically indicated risk groups need to be prioritized in infectious disease outbreaks, as it happened in Austria’s response to COVID-19. However, public health messages and protective measures often failed to match with the diverse living and working contexts of these groups and their stakeholders by defining vulnerability too uniformly. Certain groups that were not officially defined as vulnerable, as e.g. socially deprived or persons with cognitive impairments, faced difficulties in coping with epidemic regulations as well as with accessing and processing health information.

Our findings demonstrate that provisions to protect official risk groups (primary vulnerability) were part of Austria’s pandemic management approach but need to be reviewed in terms of accuracy and practicability. In contrast, secondary and tertiary levels of vulnerability remained largely unaddressed and require a process of raising awareness at political level as well as among representatives of these vulnerable populations.

To achieve durable participation of vulnerable groups and alignment of epidemic response with their diverse living conditions, change has to take place on several levels. First, decision-makers and public health authorities need to recognize the challenges of these groups and admit more self-determination to them, both in the implementation of public health regulations and in the creation of autonomous response strategies. Second, there should be awareness that participation requires investments in the production of knowledge and in the establishment of long-term collaboration structures with vulnerable groups. Aside from financial grants and additional human resources, the inclusion of vulnerable groups needs to be embedded in institutional structures, which is still not the case in Austria. So far, contacts between people in vulnerable situations and the political level happen sporadically, often disregarding requirements of accessibility and simple language.

The conclusion of the CAVE project and our recommendation is to increase persuasion on the political level as well as with stakeholders representing vulnerable groups to use the CAVE model for the creation of more inclusive protective mechanisms for future outbreaks. This process has started with presentations of our findings at discussion fora provided by the Austrian Ministry of Social Affairs, Health, Care and Consumer Protection and will continue with workshops to discuss with stakeholders the importance of including secondary and tertiary vulnerabilities in their approach. By demonstrating the potential of the CAVE model and our progress achieved, we want to contribute to the creation of permanent advocacy units on national, regional, and local government levels, where vulnerable groups and their stakeholders are represented and consulted, and inclusive dialogues on epidemic response can take place. In addition, access to scientific findings on community engagement and different formats of engagement should be made available to a larger audience in order to create public awareness of the benefits of adaption and co-creation of epidemic preparedness and response.

## Data Availability

Results and publications of the CAVE project are freely available from the project website (https://www.meduniwien.ac.at/web/forschung/projekte/cave/cave/). Access to the GIS web map can be provided upon request.

## Figure citation

The legends and figures in this article were produced by the author (P.G.) under the courtesy of the Medical University of Vienna, which holds the copyrights.

Key points

- CAVE is a project that investigated vulnerabilities and possibilities of community engagement during epidemics.
- The CAVE project accounts for an inclusive definition of vulnerability beyond medical and physical indicators.
- The CAVE model offers a guide to include vulnerable populations in a public health dialogue and in the co-creation of adapted epidemic preparedness measures.
